# Male mortality rates mirror mortality rates of older females

**DOI:** 10.1038/s41598-019-47111-w

**Published:** 2019-07-22

**Authors:** Peter Lenart, Daniela Kuruczova, Peter K. Joshi, Julie Bienertová-Vašků

**Affiliations:** 1 Department of Pathological Physiology, Faculty of Medicine, Masaryk University, Kamenice 5, building A18, 625 00 Brno, Czech Republic; 2 Research Centre for Toxic Compounds in the Environment, Faculty of Science, Masaryk University, Kamenice 5, building A29, 625 00 Brno, Czech Republic; 3 Centre for Global Health Research, Usher Institute for Population Health Sciences and Informatics, University of Edinburgh, EH8 9AG Edinburgh, UK

**Keywords:** Ageing, Epidemiology

## Abstract

Women on average live longer than men, which seems to suggest that women also age slower than men. However, the potential difference in the pace of aging between the sexes is a relatively controversial topic, and both positions, i.e. “men age faster” and “men and women age at the same pace”, have found some support. We therefore employ parametric models previously established in model organisms as well as two nonparametric approaches to compare the pace of aging between the sexes using freely available mortality data from 13 high-income countries. Our results support the hypothesis that men age faster than women while also suggesting that the difference is small and that from a practical standpoint male mortality rates behave similarly to the mortality rates of women approximately eight years their senior.

## Introduction

The life expectancies of men and women are widely recognized as being different: women worldwide live longer than men^1^. This logically leads to the question whether women also age slower than men. Both “yes” and “no” answers have found some support^2–4^. The classical argument against the notion that women age slower is the fact that men experience higher mortality rates at almost every age, i.e. that the reason for their shorter lifespan is that men are the less “robust” sex and as such exhibit higher background mortality^2,4^. On the other hand, researchers suggesting that women age slower than men note that this line of reasoning may not be altogether valid since men die from different causes at different ages^3^. Regardless of theoretical arguments, aging can be defined as an age-dependent increase in mortality^3,5,6^ and the pace of aging of men and women may therefore be empirically calculated using available mortality data an approach we employed in this article.

In addition to above-mentioned research from the field of gerontology, the longer lifespan of women has been a focus of demographic research since the mid-18^th^ century^7^. Various explanations have been suggested, including biological factors, risk acquired through the social roles, environmental conditions and behavior^8,9^. And while the search for a precise answer to the question „what is the cause of a longer female lifespan?” continues, it seems that a great portion of female lifespan advantage is caused by lifestyle choices. This is supported by a data showing that societies in which lifestyles of men and women are more homogenous than in general population have much smaller lifespan difference between sexes, examples of such populations are catholic religious orders^9^, kibbutzes^10^, Seventh-Day Adventists^11^, and others^12–14^. Accordingly, men have higher variability in mortality than women^7^. Nevertheless, even in the case of e.g. Catholic religious orders women still live longer than men^9^.

One method of quantifying aging relies on calculating the rate at which mortality increases with age^15^. The relationship between human age and mortality is usually modeled using a predefined distribution which explicitly defines the relationship between age and mortality rate. Distributions most commonly used for this purpose include Gompertz, its extension Gompertz–Makeham, Weibull or logistic^16^. The choice of a specific distribution depends on the purpose of its use: the best-fitting model is often desired when a prediction is sought while a different model may be more suitable for the interpretation of parameter values^17,18^. Since our objective was to test the difference between the pace of mortality rate increase in men and women, the Gompertz model^19^ was selected as a simple and suitable option. In addition to accommodating human mortality data between approximately 30 and 80 years of age^18,20^, it also offers a means for comparing mortality rate increase by means of mortality rate doubling time (MRDT), a parameter commonly used as an estimate of the rate of aging^21,22^. On the other hand, it does not distinguish between intrinsic and extrinsic mortality rates, where intrinsic mortality is assumed to be the result of aging and increases over time while extrinsic mortality is assumed to be caused by environmental hazards and is thus constant over time^23^. This inability to distinguish between intrinsic and extrinsic mortality rates is to some extent alleviated by the fact that mortality within the chosen interval of 30 to 60 years of age is mainly influenced by intrinsic causes^18^. However, even though the extrinsic causes are responsible for a minority of deaths within the chosen interval, they still affect the overall mortality rates. The Gompertz–Makeham model extends the Gompertz model to include mortality rate independent of age. This partitioning of mortality rates into an age-related and a constant component is clearly helpful when analyzing the rates of aging.

In this study we used mortality data obtained from the Human Mortality Database^24^ to calculate MRDTs using the Gompertz and Gompertz–Makeham model for male and female populations in 13 high-income countries. Furthermore, we have also employed two non-parametric approaches to compare the pace of aging between sexes. However, it must be said that mortality rates are affected by a great variety of external influences unrelated to aging. One extreme example of such external influences was undoubtedly World War II, which dramatically altered mortality rates both directly through the deaths of millions of soldiers and civilians and indirectly through the late effects of injuries, starvation, psychological trauma, etc. It is known that mortality rates during the early life of a cohort influence its mortality rates later in life^25^ which makes cohorts affected by a WWII unsuitable for comparing the pace of aging between sexes. Because most countries in the Human Mortality Database were more or less heavily involved in WWII, we analyzed mortality patterns only in people born at least five years after the end of this conflict. To be more specific, we analysed cohorts of people born from 1950 to 1954 using cohort mortality rates in periods starting from 1980 to 1984 to the newest available data in the Human Mortality Database. In other words, investigated mortality rates were calculated using periods starting with the subjects’ 30^th^ birthdays and ending with the end of records.

## Methods

Mortality rate data were acquired from www.mortality.org on 12 July 2017. The Human Mortality Database (HMD) contained data about mortality rates for 39 sovereign countries and several others smaller areas and populations. In our analysis we focused on 13 high-income, western (plus Japan), stable countries with populations exceeding 8 million. The analysed countries are: Australia, Belgium, Canada, France, Italy, Japan, the Netherlands, Portugal, Sweden, Switzerland, the United Kingdom, the United States of America and West Germany. The reasoning behind the selection criteria was to analyse only the most stable countries with a high population to minimalize an effect of chance and aging unrelated effects on the outcome of our analysis. Mortality data analysed for each country were in periods starting from 1980 to 1984 to the newest available data in the Human Mortality Database in most cases this was the year 2014. However, for the UK, Italy, and Canada it was 2013, 2012 and 2011 respectively.

### Gompertz and Gompertz-Makeham model

The Gompertz model^19,21^ of exponential hazard growth was used to model the relationship between age and mortality rate. The basic form of the Gompertz model is

$$h(t)=a{e}^{bt}$$

where *a* and *b* are constants, *t* is time (age), and *h(t)* is the hazard (mortality) rate. Using the logarithmic transformation, a simple linear model is obtained

$$\mathrm{log}\,h(t)=\,\mathrm{log}(a)+bt.$$

where *log(a)* signifies the intercept (overall shift of the line in the direction of the y-axis) and *b* expresses the slope of the line. MRDT is subsequently calculated from the slope as

$$MRDT=\frac{\mathrm{log}(2)}{b}$$

and expresses the time it takes for the mortality rate to double.

The Gompertz–Makeham model is a natural extension of the Gompertz model obtained by adding a constant^26^:

$$h(t)=c+a{e}^{bt}.$$

The constant *c* expresses the part of mortality that does not depend on age. Focusing only on the age-dependent part of the equation, the mortality rate doubling time can be obtained in the same manner as in the Gompertz model, using the value of parameter *b*.

We fitted the above described on data for each individual country using an age interval beginning at 30 years of age. To distinguish between male and female models, we added a dummy variable for sex and fitted the following model:

$$h(t)=(c+{\Delta }c)+(a+\Delta a){e}^{(b+{\Delta }b)t},$$

where *Δa*, *Δb*, and *Δc* signify the difference between parameter values in males and females. These values were subsequently tested against zero (i.e. there is no difference in the parameter between males and females). P-values are only informative, no conclusions are drawn from them because of small statistical power. Due to the exponential nature of models, we used numerical fitting via non-linear least squares. Both models accurately fit human mortality dynamics roughly between 30 and 80^18,20^, which we subsequently confirmed using an exploratory analysis of HMD data.

### Nonparametric approaches

#### Smoothing spline approach

We also used a nonparametric approach to model the relationship between age and mortality rate. We approximated the death rate data by a cubic smoothing spline. Cubic smoothing splines are commonly used to obtain underlying function from noisy data while putting no assumptions on the estimated function. Smoothing parameter controls the smoothness of the resulting curve^27^. The smoothing parameter of 0.7 was selected by visual inspection. Since the cubic spline estimate consists of polynomials of the third degree, we are able to calculate a derivative curve. The value of the derivative at each time point expresses the rate of mortality increase. The derivative value would be zero for a constant mortality rate, positive values indicate an increase in the mortality rate over time – the higher the derivative value, the steeper the mortality curve.

#### Mortality rate matching approach

To gain more intuitive insight into the difference between male and female mortality rates we matched the female mortality rates with male mortality rates. To ensure the continuity of both male and female mortality rates over time, we used mortality rate curves obtained using the previous approach. The resulting line expresses the ages where the female mortality rate equals the male mortality rate. Compared males and females were always born in the same year and country.

#### Aging as a derivate of mortality on natural or log-scale

It is important to acknowledge that the mortality can be assessed on two different scales – the natural and logarithmic one. We made a conscious decision not to fix on either scale. We present the Gompertz and Gompertz-Makeham model as approaches based on the log scale mortality and the derivative ratio as natural scale approach. The last presented method of mortality matching is scale-assumption free as it yields the same results for both of them. Obviously, the derivative ratio approach could be used on log scale data as well. We do not present these results as they correspond to the Gompertz model results, as is shown in Figs S1 and S2 in the supplementary material. We also explored the possibility that Gompertz/Gompertz Makeham model holds when using the derivative ratio and mortality matching approaches. Even with this assumption, the conclusions would be very similar as supported by a reasoning and mathematical calculations provided in the supplementary methods. That essentially means that even when Gompertz/Gompertz Makeham model holds and we use derivative ratio or mortality matching approach, the ultimate conclusion on whether males age faster or not, is in the majority of cases the same as it would be in Gompertz/Gompertz Makeham model. However, while the direction of the difference will be the same, its magnitude may vary.

## Results

### Gompertz model

MRDTs calculated for people born in 1954 are longer for males in 10 out of 13 countries (Fig. S1 and Table S1). However, the possibility of longer male MRDTs is inconsistent with MRDTs calculated for 1953, 1952, 1951 and 1950 cohorts. Males born in 1953 have longer MRDTs in 7 out of 13 countries but those born in 1952 only in 6 out of 13. Furthermore, males born in 1951 and 1950 have longer MRDTs only in 8 and 7 countries respectively. Gompertz model results thus suggest that MRDTs are the same for males and females.

### Gompertz–Makeham model

**Figure 1:**
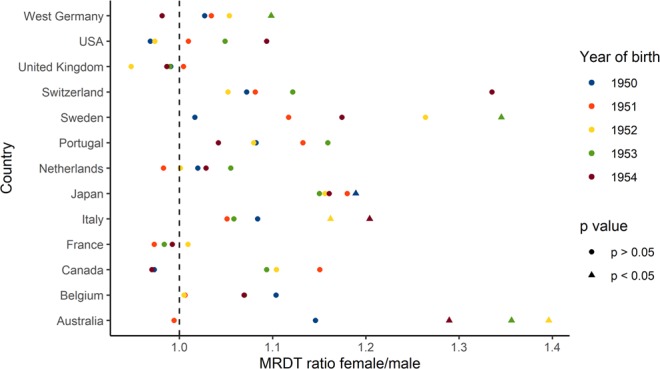
Mortality rate increase is greater for men than women. The figure provides a summary of the ratio between female and male MRTDs calculated using the Gompertz–Makeham model for all analyzed countries and birth cohorts.

Contrary to the results of the Gompertz model, MRDTs calculated using the Gompertz–Makeham model for 1950–1954 cohorts exhibit consistent differences between the sexes (Fig. 1 and Table S2). MRDTs for the 1954 cohort are longer for women in 9 out of 13 countries while MRDTs for the 1953 cohort are longer for women in 11 countries. This trend is further evident in all remaining cohorts. MRTDs for the 1952, 1951 and 1950 cohorts are higher for females in 11, 10 and 9 out of 13 countries respectively.

**Figure 2:**
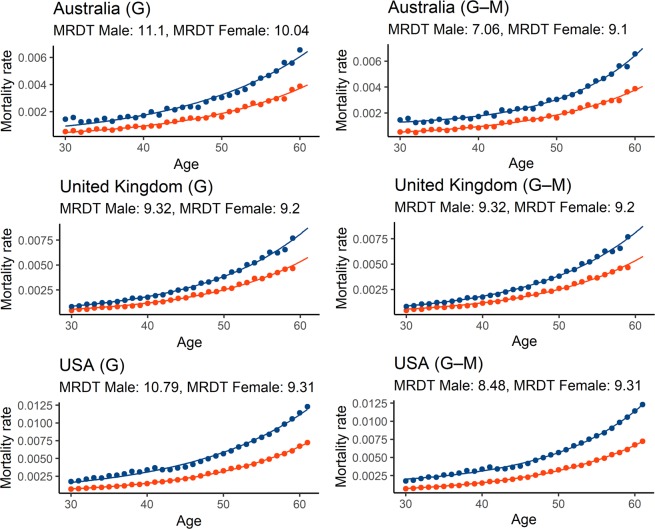
Gompertz and Gompertz–Makeham models provide similar fit. (A) Comparison between curve shapes for Gompertz (G) and Gompertz Makeham (G–M) in three selected countries. Blue represents male and red female mortality curves.

The disparate outcomes of the Gompertz and Gompertz–Makeham models are most likely caused by different parametrization rather than by different curve shapes (Fig. 2). When the age-independent parameter c is not included, like in the Gompertz model, the value of the other two parameters changes accordingly in order to provide a best-fitting curve. If a roughly similar mortality curve were to be described by the Gompertz and Gompertz–Makeham models, the following would apply: the growing c value of the Gompertz–Makeham corresponds to a decreasing b value in the Gompertz model and thus an increasing MRDT. We have found that if the age-independent parameter c is non-zero, it is higher in males (Table S2), which partially explains the different results achieved using the Gompertz and Gompertz–Makeham models. If the value of the parameter is zero, the model reduces to Gompertz and provides the same results. It is also important to note that almost all cases where the Gompertz–Makeham model reduces to Gompertz have either longer MRDT in females or almost negligible difference between MRDTs.

### Smoothing spline approach

**Figure 3:**
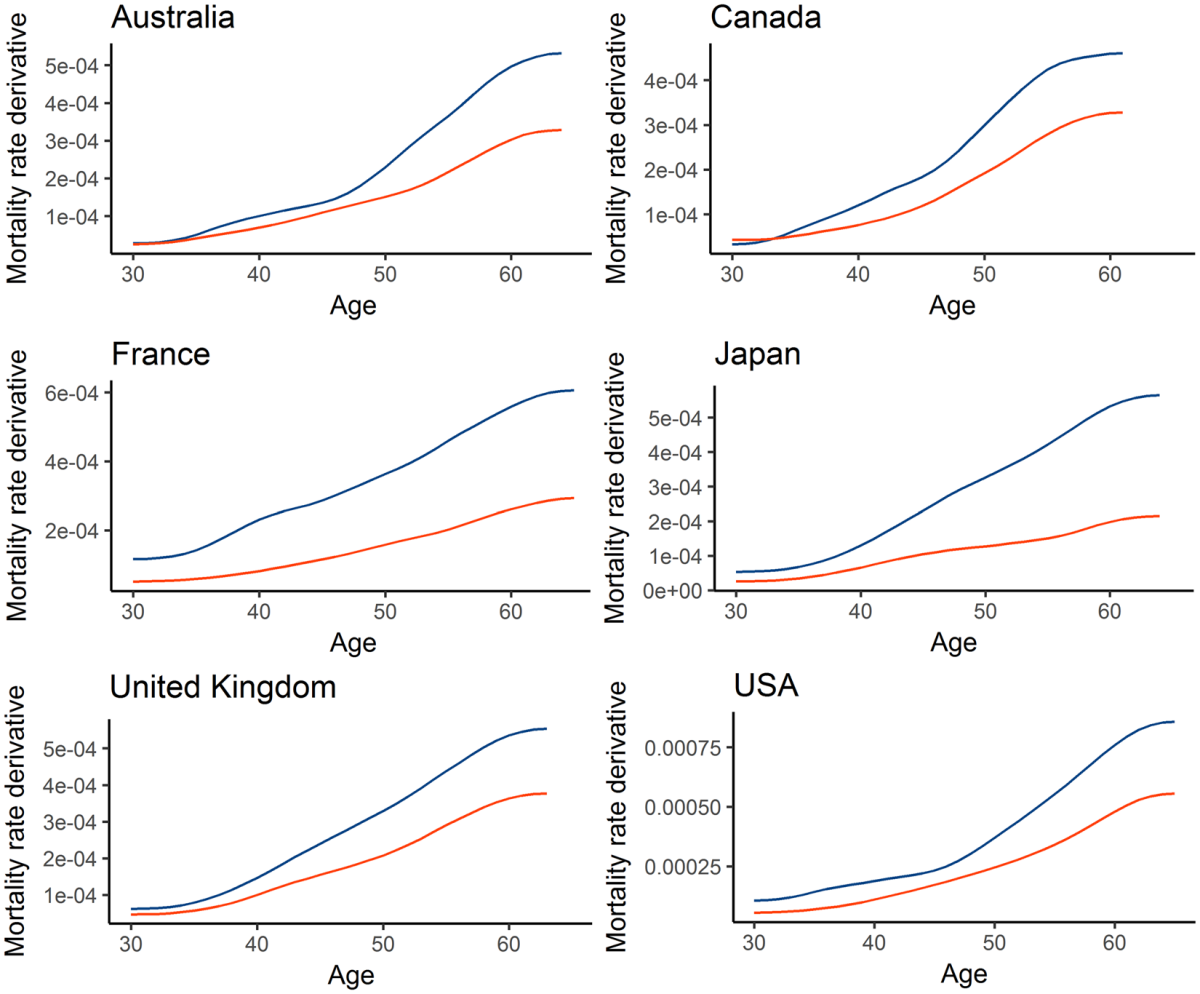
Mortality curves of men are steeper than those of women. The figure shows derivative curves obtained for men and women using a nonparametric approach for six selected countries in the 1950 cohort. Blue mortality curves are for men while red for female.

We employed a nonparametric approach to further test the difference in the pace of aging between the sexes without the constraint of parametric models. Results in the form of derivative curves, where the value of the derivative at each time point constitutes an increase of mortality rate per year, (Fig. 3 and Table S3) clearly show that mortality rates increase faster in men than in women in all studied countries and cohorts. These results are thus in agreement with the results of the Gompertz–Makeham model.

**Figure 4:**
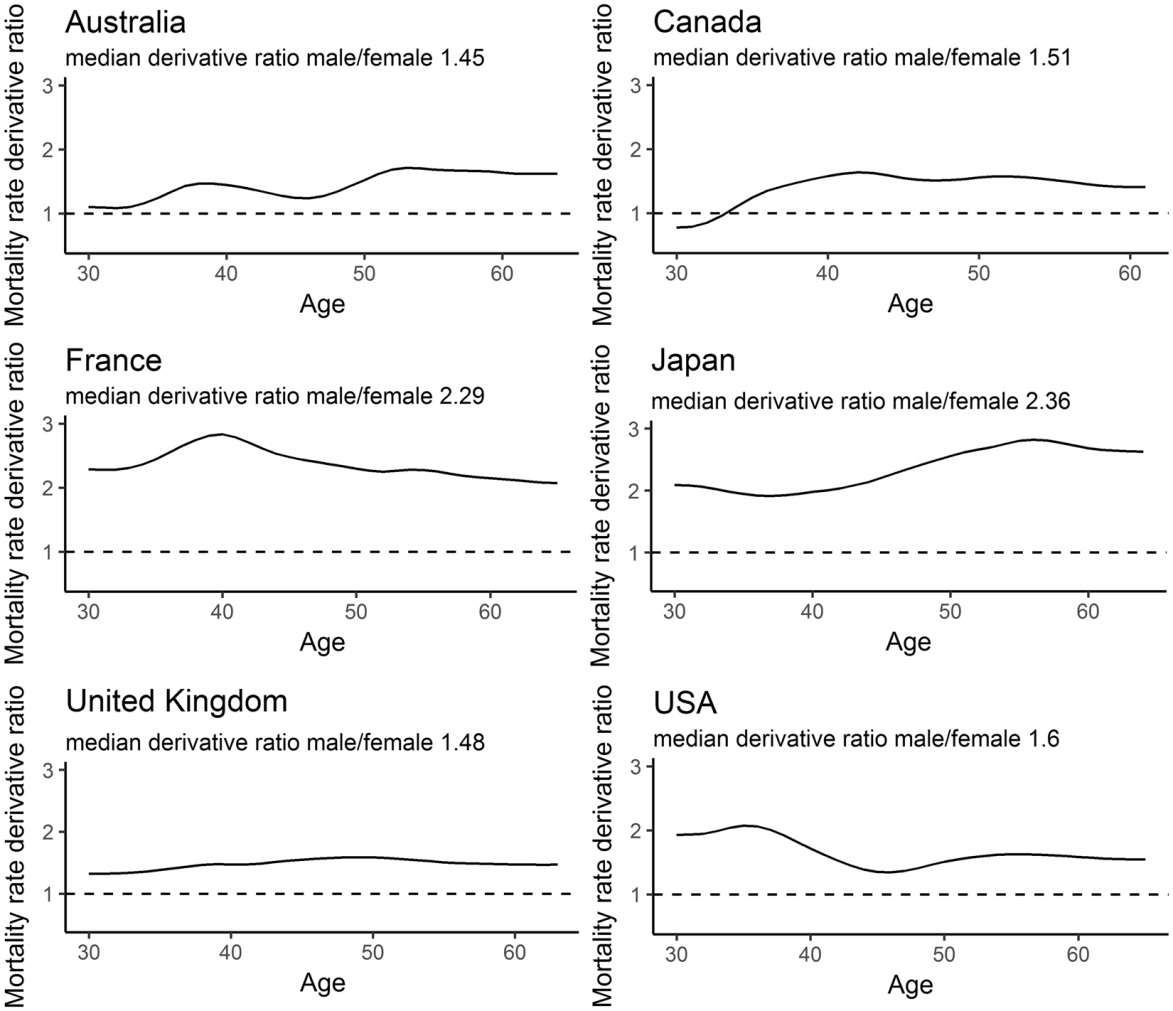
Quantitative differences in male and female mortality rate increase. The figure shows male/female death rate derivative ratios for 1950 in six selected countries.

**Figure 5:**
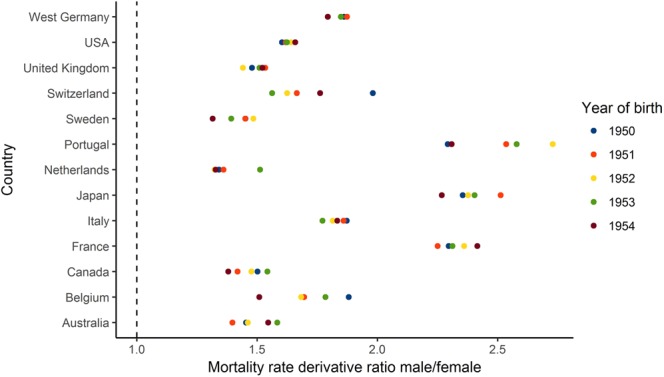
Nonparametric approach shows universally higher increase in male mortality. The figure shows male/female derivative rations for all studied countries and cohorts.

Furthermore, a comparison of derivative curves for males and females enables us to quantify the ratio of annual male and female mortality rate increase (Fig. 4). The median derivative ratio for males and females ranges from 1.34 in the Netherlands to 2.36 in Japan (Fig. 5). In other words, the median increase in male mortality rates for this cohort is 34 to 136% higher than that in females. The median derivative ratio for the 1951 cohort ranges from 1.37 for the Netherlands to 2.56 for Portugal. The ranges for 1952, 1953 and 1954 cohorts are 1.28–2.68, 1.33–2.87, and 1.3–2.38 respectively.

### Mortality rate matching approach

**Figure 6:**
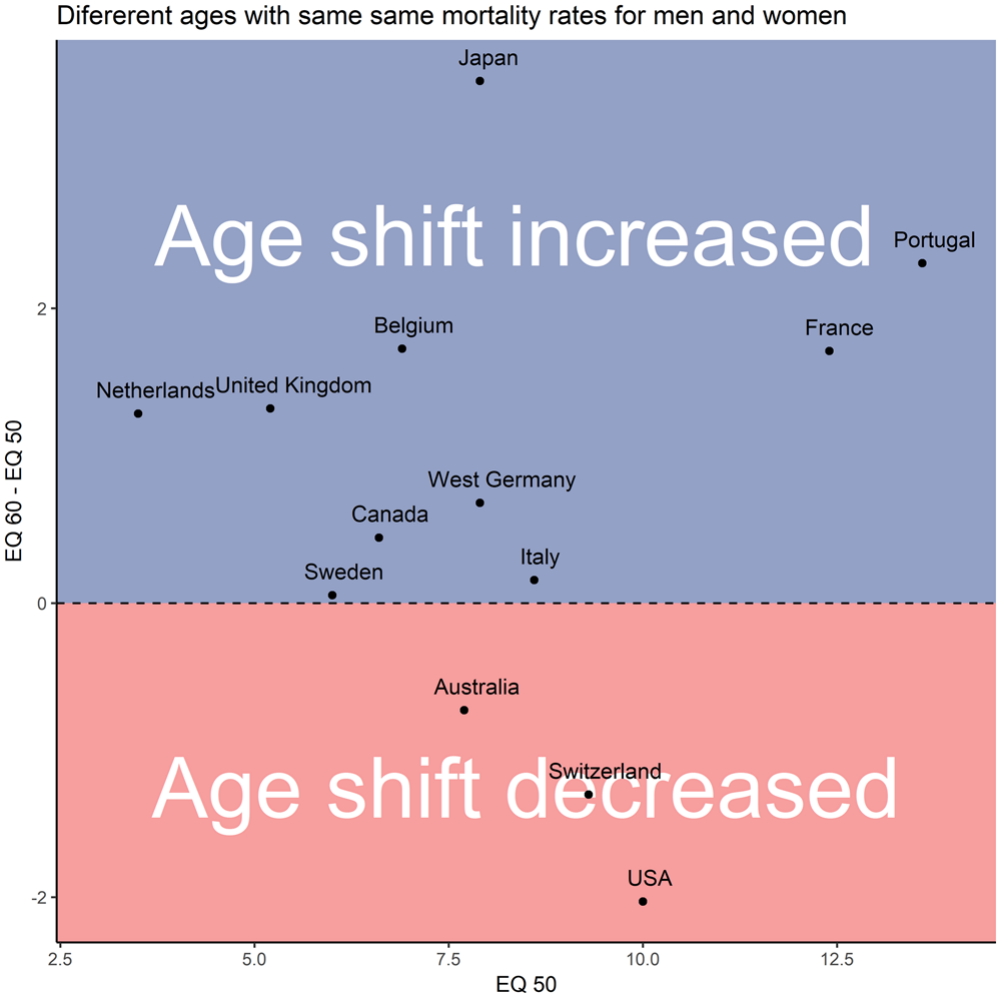
Men achieve mortality rates equal to that of women aged 50 many years earlier. Equivalent mortality (EQ) at age X expresses how much earlier men achieve the same mortality rates as women aged X. For example, an EQ. 50 of 10 means that men aged 40 exhibit the same mortality rates as women aged 50. The y axis captures how differences in age with the same mortality rates high-income over a ten-year-long period in the studied countries and cohorts.

Since the selected calculation methods may have influenced our results, we have decided to employ a second nonparametric approach. By comparing absolute mortality rate values rather than derivatives, we tested whether our results are robust enough to withstand various methodological approaches. When comparing the mortality rates of males and females, we found that the mortality rates of females aged 50 are on average equal to the mortality rates of males aged 41.9 years (Fig. 6). The smallest age gap was in Netherlands where men attained the mortality rates of 50 years old women in 46.53 years. The widest gap was in Portugal, where 36.39 years old males already had mortality rates of 50 years old females. When we made the same comparison for the mortality rates of women aged 60 we found out that men had already achieved equal mortality rates at the average age of 51.2. In other words, the gap between female and male mortality rates increased on average by 0.7 years over the course of the decade (Fig. 6), suggesting that males age faster.

**Figure 7:**
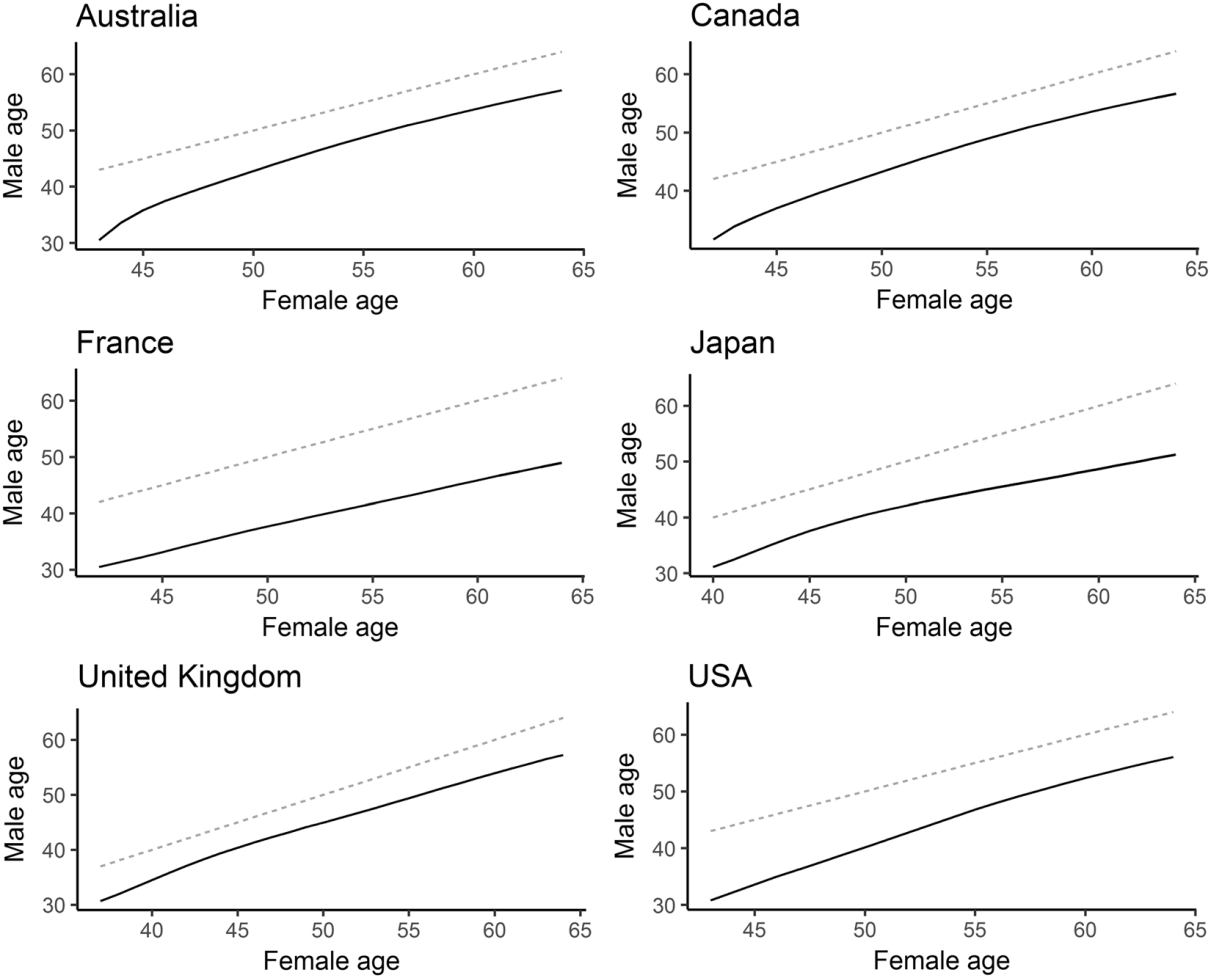
Male mortality rates behave like mortality rates of females several years older. The bottom line in each graph connects ages at which males and females exhibit identical mortality rates. The dotted lines represent a hypothetical situation where males exhibit the same mortality rates as females of the same age.

On the other hand, when we compared the age difference between equal male and female mortality rates using longer timescales, it seems that the observed changes are rather small and that male mortality rates have a dynamic similar to the mortality rates of females several years older (Fig. 7).

## Discussion

We investigated mortality data to test whether men age faster than women, as previously suggested in several studies^3,28–30^. While calculations of MRDTs conducted using the Gompertz model do not show a consistent difference in the pace of aging between the sexes, the results of the Gompertz–Makeham suggests that men age faster than women. The difference between the results of the Gompertz and Gompertz–Makeham model may be partially explained by the fact that, unlike the Gompertz model, the Gompertz–Makeham model includes an age-independent parameter c which is higher in men. Thus, in Gompertz model age-independent mortality attributes to MRDTs and inflates male MRDTs more than female MRDTs, which implies that the Gompertz–Makeham model is better suited to comparing aging between the sexes.

Nevertheless, although the Gompertz and Gompertz–Makeham models are certainly useful, they are ultimately highly constrained models which may sometimes produce incorrect results. We therefore also compared the pace of aging between the sexes using non-parametric approaches. The results of the smoothing spline approach suggest that men age faster than women, i.e. they are in agreement with with the outcome of the Gompertz–Makeham model. On the other hand, the results of the mortality matching rates approach show that while men may age faster on average, the difference between the male and female paces of aging is rather small. Furthermore, the results of the mortality matching rates approach show that male mortality rates behave similarly to the mortality rates of females approximately eight years older. Taken together, our results suggest that while men may age faster than women, the difference in the pace of aging between sexes is rather small and men mortality rates behave mostly as mortality rates of older women.

While our analysis of mortality data does not distinguish between intrinsic and extrinsic sources of mortality, this partitioning has been examined in several existing studies^31,32^. Despite the fact that mortality partitioning remains a gold standard which may help bring important insight into the aging process in many situations, it is in fact rather superficial: the assumption that intrinsic mortality sources are caused by aging while extrinsic mortality sources are caused by environmental influences – and are thus constant over time – is simply wrong^23^. Accordingly, even Bruce A. Carnes and S. Jay Olshansky, arguably the two most influential authors studying mortality partitions, sharply disagree with this naive assumption. This is probably best documented by the fact that both are among the authors of a paper which clearly states that “It is difficult to envision a cause of death for humans or any other species, either intrinsic or extrinsic, that does not exhibit age-dependence^31^”. Furthermore, it is documented that even mortality caused by accidents such as falls, drowning, transport accidents and exposure to mechanical forces dramatically increases with age, as does the number of deaths caused by natural disasters, including excessive heat or cold, earthquakes, lightning, storms, and floods^23^. In other words, biologically older individuals are at a higher risk of death from both intrinsic and extrinsic sources. We thus believe that using overall i.e., non-partitioned mortality to compare the pace of aging should be sufficient or even preferable to focusing purely on intrinsic mortality.

The rates of aging calculated by different approaches in this article show a large variability across the analysed countries; this implies that pace of aging calculate in this manner may be affected by external factors, e.g., social and lifestyle factors. Therefore, the precise values of our results have to interpret with caution. Nevertheless, the results across the different countries show consistent trends, and we believe they are sufficient to answer question of whether men age faster than women qualitatively.

Overall, our study demonstrates that a comparison of the paces of aging may yield vastly different results when different methods are employed (e.g., Gompertz vs. Gompertz–Makeham), which is an issue deserving of broader scientific attention. Furthermore, our results show that if men and women age at different paces, the difference is rather small and it seems that, from a practical viewpoint, male mortality rates behave in the same way as the mortality rates of women several years older.

## Supplementary information

Supplementary figure 1

Supplementary figure 2

Supplementary table 1

Supplementary table 2

Supplementary table 3

supplementary methods

## Data Availability

Code is available at http://www.math.muni.cz/~xkuruczovad/Mortality/ or upon request.

## References

[1] Barford A, Dorling D, Smith GD, Shaw M (2006). Life expectancy: women now on top everywhere. BMJ.

[2] Austad SN (2006). Why women live longer than men: Sex differences in longevity. Gend. Med..

[3] Blagosklonny MV (2010). Why men age faster but reproduce longer than women: mTOR and evolutionary perspectives. Aging.

[4] Austad SN, Fischer KE (2016). Sex Differences in Lifespan. Cell Metab..

[5] Kirkwood TBL, Austad SN (2000). Why do we age?. Nature.

[6] Lenart P, Bienertová-Vašků J (2017). Keeping up with the Red Queen: the pace of aging as an adaptation. Biogerontology.

[7] Luy M, Gast K (2014). Do Women Live Longer or Do Men Die Earlier? Reflections on the Causes of Sex Differences in Life Expectancy. Gerontology.

[8] Wingard DL (1984). The sex differential in morbidity, mortality, and lifestyle. Annu. Rev. Public Health.

[9] Luy M (2003). Causes of Male Excess Mortality: Insights from Cloistered Populations. Popul. Dev. Rev..

[10] Leviatan U, Cohen J (1985). Gender differences in life expectancy among Kibbutz members. Soc. Sci. Med..

[11] Berkel J, de Waard F (1983). Mortality pattern and life expectancy of Seventh-Day Adventists in the Netherlands. Int. J. Epidemiol..

[12] Miller GH, Gerstein DR (1983). The life expectancy of nonsmoking men and women. Public Health Rep..

[13] Staetsky LD, Hinde A (2009). Unusually small sex differentials in mortality of Israeli Jews: What does the structure of causes of death tell us?. Demogr. Res..

[14] Hamman RF, Barancik JI, Lilienfeld AM (1981). Patterns of mortality in the the Old Order Amish. I. Background and major causes of death. Am. J. Epidemiol..

[15] Pletcher SD, Khazaeli AA, Curtsinger JW (2000). Why do life spans differ? Partitioning mean longevity differences in terms of age-specific mortality parameters. J. Gerontol. A. Biol. Sci. Med. Sci..

[16] Pham, H. Mortality Modeling Perspectives. *In Recent Advances in Reliability and Quality in Design* 509–516 (Springer, London), 10.1007/978-1-84800-113-8_25 (2008).

[17] Ricklefs RE, Scheuerlein A (2002). Biological Implications of the Weibull and Gompertz Models of Aging. J. Gerontol. Ser. A.

[18] Nash, F. R. *Reliability Assessments: Concepts, Models, and Case Studies*. (CRC Press, 2016).

[19] Gompertz B (1825). On the Nature of the Function Expressive of the Law of Human Mortality, and on a New Mode of Determining the Value of Life Contingencies. Philos. Trans. R. Soc. Lond..

[20] Easton DM, Hirsch HR (2008). For prediction of elder survival by a Gompertz model, number dead is preferable to number alive. Age.

[21] de Magalhães JP, Cabral JAS, Magalhães D (2005). The Influence of Genes on the Aging Process of Mice. Genetics.

[22] de Magalhães JP, Costa J, Church GM (2007). An Analysis of the Relationship Between Metabolism, Developmental Schedules, and Longevity Using Phylogenetic Independent Contrasts. J. Gerontol. A. Biol. Sci. Med. Sci..

[23] Koopman JJE, Wensink MJ, Rozing MP, van Bodegom D, Westendorp RGJ (2015). Intrinsic and extrinsic mortality reunited. Exp. Gerontol..

[24] Human Mortality Database. Available at, http://www.mortality.org/ (Accessed: 30th June 2017).

[25] Beltrán-Sánchez H, Crimmins EM, Finch CE (2012). Early Cohort Mortality Predicts the Cohort Rate of Aging: an Historical. Analysis. J. Dev. Orig. Health Dis..

[26] Pletcher (1999). Model fitting and hypothesis testing for age-specific mortality data. J. Evol. Biol..

[27] de Boor, C. *A Practical Guide to Splines*, Volume 27 (1978).

[28] Barrett ELB, Richardson DS (2011). Sex differences in telomeres and lifespan. Aging Cell.

[29] Phillip JM (2017). Biophysical and biomolecular determination of cellular age in humans. Nat. Biomed. Eng..

[30] Kolovou Genovefa D., Kolovou Vana, Mavrogeni Sophie (2014). We Are Ageing. BioMed Research International.

[31] Carnes BA, Holden LR, Olshansky SJ, Witten MT, Siegel JS (2006). Mortality Partitions and their Relevance to Research on Senescence. Biogerontology.

[32] Olshansky SJ, Carnes BA (1997). Ever since Gompertz. Demography.

