# Supplementary material for: Male mortality rates mirror mortality rates of older females
Source: Sci Rep. 2019 Jul 22;9:10589. doi: 10.1038/s41598-019-47111-w (PMC6646351; doi:10.1038/s41598-019-47111-w)
Supplement: Supplementary file 2 — Supplementary figure 2 [file 41598_2019_47111_MOESM2_ESM.pdf]

Male mortality rates mirror mortality rates of older females

Authors

Peter Lenart[1,2*#], Daniela Kuruczova[1#], Peter K. Joshi[3], Julie Bienertová-Vašků[1, 2]

The authors contributed to the manuscript in equal measure.

[1]*Department of Pathological Physiology, Faculty of Medicine, Masaryk University, Kamenice 5, building A18, 625 00, Brno, Czech Republic*

[2]*Research Centre for Toxic Compounds in the Environment, Faculty of Science, Masaryk University, Kamenice 5, building A29, 625 00, Brno, Czech Republic*

[3]*Centre for Global Health Research, Usher Institute for Population Health Sciences and Informatics, University of Edinburgh, Edinburgh, EH8 9AG, UK*

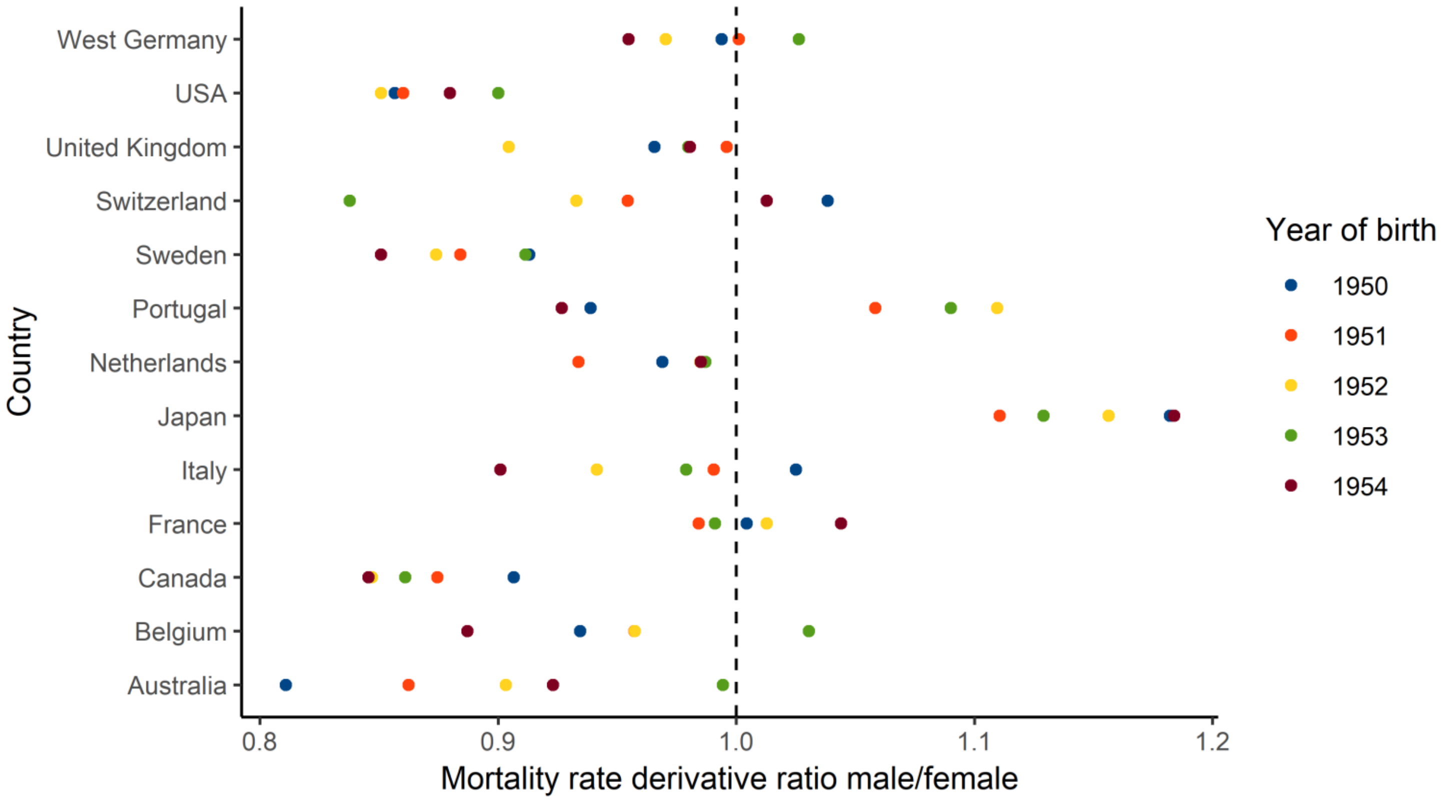

West Germany
USA
United Kingdom
Switzerland
Sweden
Portugal
Netherlands
Japan
Italy
France
Canada
Belgium
Australia
Country
0.8
0.9
1.0
1.1
1.2
Mortality rate derivative ratio male/female
Year of birth
1950
1951
1952
1953
1954
