# Supplementary material for: Male mortality rates mirror mortality rates of older females
Source: Sci Rep. 2019 Jul 22;9:10589. doi: 10.1038/s41598-019-47111-w (PMC6646351; doi:10.1038/s41598-019-47111-w)

Male mortality rates mirror mortality rates of older females

Authors

Peter Lenart[1,2*#], Daniela Kuruczova[1#], Peter K. Joshi[3], Julie Bienertová-Vašků[1, 2]

The authors contributed to the manuscript in equal measure.

[1]*Department of Pathological Physiology, Faculty of Medicine, Masaryk University, Kamenice 5, building A18, 625 00, Brno, Czech Republic*

[2]*Research Centre for Toxic Compounds in the Environment, Faculty of Science, Masaryk University, Kamenice 5, building A29, 625 00, Brno, Czech Republic*

[3]*Centre for Global Health Research, Usher Institute for Population Health Sciences and Informatics, University of Edinburgh, Edinburgh, EH8 9AG, UK*

Derivative ratio approach on the natural scale while Gompertz/Gompertz-Makeham Model holds

We will base our reasoning on the assumption that the Gompertz-Makeham (G-M) model holds as the Gompertz model is just a special case of the G-M model.

We assume that G-M model holds and the mortality rate can be expressed as follows:

$$m(Age) = c + e^{a+b.Age}.$$

Then it is straightforward to obtain a formula for derivative ratio for male mortality and female mortality

$$\frac{m_M{}'(Age)}{m_F{}'(x)} = \frac{b_M}{b_F}\; e^{(b_M-b_F)Age} e^{(a_M-a_F)}$$

where the index M denotes the male mortality rate and model parameters and the index F the female ones. Thanks to the derivation, the Makeham term disappears, and its value does not influence the results. It is visible, that difference $b_M - b_F$ determines the steepness and direction of the resulting curve, and difference $a_M - a_F$ its vertical position. The Figure S3 illustrates these effects with parameter values close to those fitted in the G-M model (see Supplementary table 1 and 2). The difference between a parameters is very small so we decided to slightly exaggerate it to highlight the vertical shift. If we used the actual fitted values, the curves would visually overlap. Even when the difference is exaggerated, it is visible, that the change in the vertical position is almost negligible compared to the slope of the curve. To illustrate the effects on actual data, we created a derivative ratio plot for Gompertz and G-M model for Australia (cohort 1950). It is visible, that the Gompertz model curve is slightly dropping and is very close to 1, which suggests that females age slightly faster than males but the difference is small. The very same conclusion can be drawn from the Gompertz model itself (male MRDT 9.81 vs. female MRDT 9.36). The G-M curve is visibly increasing and is further apart from the 1. This suggests that the males age faster than females, which is in correspondence with the G-M model results (male MRDT 8.09 vs. female MRDT 8.79). Thus it can be concluded that even when the G-M model holds, the conclusion about the different rate of ageing is the same.

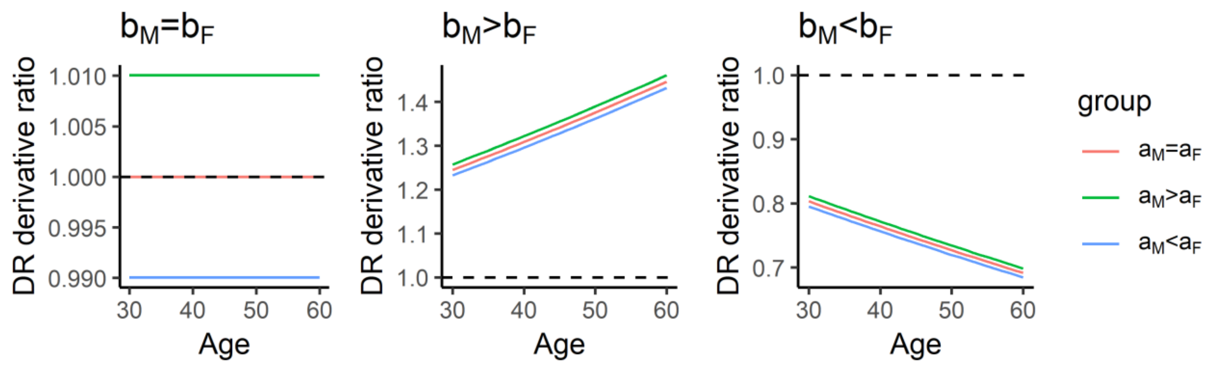

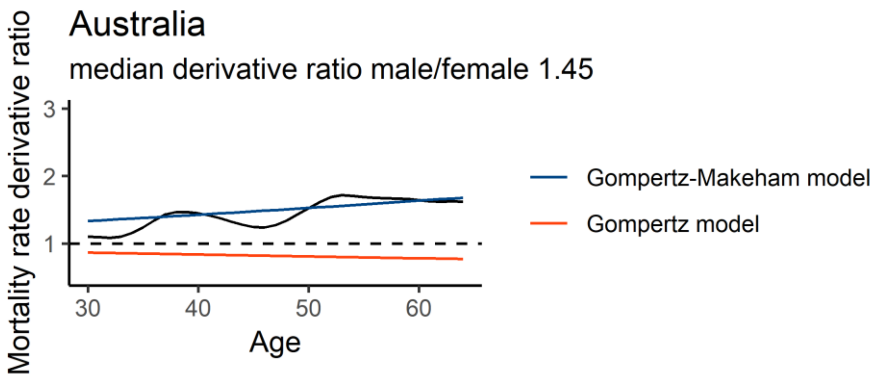

Mortality matching approach while Gompertz/Gompertz-Makeham Model holds

Mortality matching approach in the main article was based on the idea of finding male age with an equal mortality rate as a female aged X. In this case, it is considerably less straightforward to derive an exact formula for the G-M model, so we used a numerical calculation to obtain the results. However, the formula for the Gompertz is still quite easy to obtain as follows:

$$\text{EQ Age}_M = \frac{b_F}{b_M} Age_F + \frac{a_F - a_M}{b_M}.$$

It can also be used to roughly describe the Gompertz-Makeham model as is apparent from Figure S4 below. To show the difference visually, the b and c parameters have been slightly exaggerated. The difference $b_M - b_F$ controls the slope of the line, and the difference $a_M - a_F$ the vertical shift. The difference between Makeham terms $c_M - c_F$ controls whether the curve approaches the line from below of above. It is apparent, that the position below or above the $x = y$ line is mainly determined by the parameters b and a. As the effect of the vertical shift is exaggerated in the pictures, we can conclude, that using this approach, we should be able to distinguish between different rates of ageing in the G-M model.

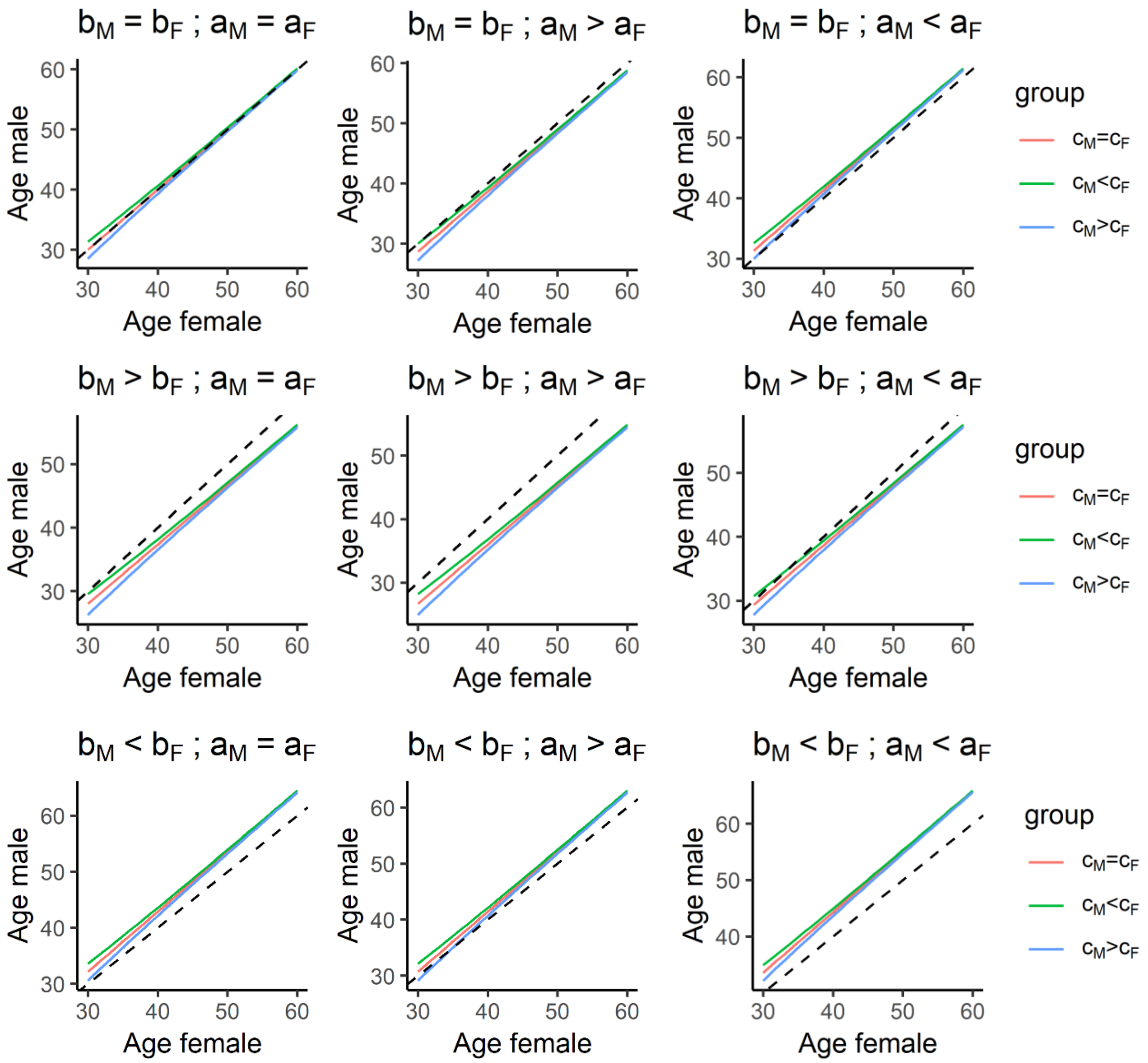

Supplement: Supplementary file 6 — supplementary methods [file 41598_2019_47111_MOESM6_ESM.pdf]
